# Comparison of mediterranean and healthy eating guideline interventions on the dietary inflammatory index in rheumatoid arthritis: results from a dietary randomised controlled intervention trial

**DOI:** 10.1007/s00394-026-03987-9

**Published:** 2026-05-26

**Authors:** Megan Curran, Nicola Canning, Ailish Wrenne, Tala Raad, James R. Herbert, Nitin Shivappa, Audrey Tierney

**Affiliations:** 1 School of Allied Health, University of Limerick, V94 T9PX Limerick, Ireland; 2 Health Research Institute, University of Limerick, V94 T9PX Limerick, Ireland; 3 Centre for Implementation Research at UL, University of Limerick, V94 T9PX Limerick, Ireland; 4 Cancer Prevention and Control Program and Department of Epidemiology and Biostatistics, Arnold School of Public Health, University of South Carolina, Columbia, SC USA; 5 Department of Nutrition, Connecting Health Innovations, LLC, Columbia, SC, USA

**Keywords:** Rheumatoid arthritis, Dietary inflammatory index, Mediterranean diet, Healthy eating guidelines

## Abstract

**Purpose:**

Anti-inflammatory diets, such as the Mediterranean Diet (MedDiet) have shown positive effects on disease activity in Rheumatoid Arthritis (RA). The Dietary Inflammatory Index (DII) has been associated with RA risk. However, the effect of improving diet quality with a MedDiet and impact on the DII and patient-reported outcome measures (PROMs) has not been investigated.

**Objectives:**

To assess the effects of a MedDiet and adherence to the Irish Healthy Eating Guidelines (HEG) on change in DII and to determine whether change in DII and energy-adjusted DII (e-DII) scores is associated with improvements in PROMs in adults with RA in Ireland.

**Methods:**

40 Participants were randomised to a MedDiet (*n* = 20) or a HEG intervention (*n* = 20) for 12 weeks. DII was calculated based on food diaries collected. Between and within group data was analysed in SPSS.

**Results:**

Baseline e-DII was 0.99 ± 2.37, 0.79 ± 2.60, 1.20 ± 2.16 for total cohort (*n* = 40), MedDiet, and HEG groups, respectively (*p* = 0.588). e-DII significantly improved for the cohort following the MedDiet (*p* = 0.022) and HEG (*p* = 0.004) groups. Differences in PROMs across tertiles of e-DII change were not statistically significant, irrespective of diet assignment. Participants in the most anti-inflammatory e-DII tertile group had significantly greater intakes of omega-3 fatty acids, dietary fibre, vitamin A, vitamin E, folic acid, and beta-carotene compared to those in the pro-inflammatory tertile group (*p* < 0.05).

**Conclusions:**

Improving dietary quality with either a MedDiet or the Irish HEG improved e-DII scores in a cohort of people living with RA, however, no statistically significant change in PROMs were observed.

## Introduction

Rheumatoid arthritis (RA) is a chronic systemic inflammatory disease primarily affecting synovial joints including hands, feet, knees and hips, causing pain, stiffness and swelling of the joints [1–3]. RA affects 1 in 200 people worldwide with an estimated 40,000 people in Ireland living with RA, and a higher prevalence in women and older people [1, 3, 4]. Chronic joint inflammation, a consequence of RA, can lead to bone erosion, disability, reduced functional ability, and impaired quality of life [5–7]. Pharmacotherapy including Disease-Modifying Anti-Rheumatic Drugs (DMARDs), non-Steroidal Anti-Inflammatories (NSAIDs) and glucocorticoids are commonly used to manage RA [8]. However, not all patients respond well or observe improvements with medications [9].

The exact aetiology is unknown, but it is believed that both genetic and external factors contribute to the autoimmune response observed in patients with RA [10]. The inflammatory process is driven by immune cells and mediators including B cells, T cells, macrophages and fibroblasts which secrete pro-inflammatory cytokines and chemokines contributing to inflammation and subsequent joint damage and pain [11].

Diet is an environmental factor with the potential to influence inflammation, RA symptoms and RA incidence [12–14]. The Western Diet rich in processed foods, red meat, refined grains and full fat dairy products contains high levels of arachidonic acid (AA), an omega 6 (n-6) polyunsaturated fatty acid that contributes to the production of pro-inflammatory eicosanoids contributing to low-grade systemic inflammation causing significant pain in patients with RA [15–16].

On the contrary, The Mediterranean Diet (MedDiet) characterised by a large consumption of fruit, vegetables, wholegrains, culinary herbs and spices, olive oil as the main source of added fat, moderate amounts of dairy, poultry and wine, and limited red meat consumption is considered anti-inflammatory [17, 18]. The anti-inflammatory properties of the MedDiet are mainly attributed to its monounsaturated fat content (particularly from extra-virgin olive oil), omega 3 fatty acid content (from oily fish) and antioxidants from plant foods, all of which reduce oxidative stress and pro-inflammatory cytokines [17, 19].

The MedDiet has been shown to benefit individuals with RA, and it is endorsed in the National Institute for Health and Care Excellence (NICE) guidelines [20].

Previous systematic reviews reported that adherence to MedDiet interventions results in significant improvements in pain and fatigue levels in patients with RA, in addition to significant reductions in biomarkers of inflammation such as interleukin (IL)-6, IL-1β and C-reactive proteins [21, 22]. Furthermore, The Mediterranean Diet Intervention in patients with Rheumatoid Arthritis (MEDRA) randomised controlled trial (RCT), the primary study upon which this present study is based, reported that the MedDiet resulted in significant improvements in physical function and quality of life [23].

Similar to other European countries, The Department of Health in Ireland has established evidence-based guidelines to promote a healthy balanced diet for individuals aged 5 and older [24]. The Irish Healthy Eating Guidelines (HEG) include resources such as the food pyramid to support key Irish HEG including recommendations to eat 5–7 portions of fruit and vegetables, 3–5 servings of wholegrain foods, and 3 servings of low-fat dairy products, milk and yoghurt. The Irish HEG also advise to consume lean meat, poultry, fish along with eggs, beans, and lentils as protein sources, use oils and spreads sparingly, opting for reduced-fat options, and favour cooking methods such as baking, steaming, or boiling over frying [24]. Limit high-calorie, sugary, and salty snacks, avoid adding salt during cooking and choose low-salt foods, stay hydrated, primarily with water and partake in regular physical activity [24].

The Dietary Inflammatory Index (DII), an index developed to describe the inflammatory potential of the diet, is also of interest when investigating diet and inflammation [25]. The DII regards the body as a whole, examining the effect of nutrient and food parameters (comprised of nutrients and bioactive compounds) on inflammatory markers (CRP, IL-1β, IL-4, IL-6, IL-10, and tumour necrosis factor-α (TNF-α)) in the body [26]. The DII ranges from maximally anti-inflammatory (lowest negative result) to maximally pro-inflammatory (highest positive result) values [25]. DII has been associated with various health outcome measures in other chronic diseases including cardiovascular disease, cancer and all-cause mortality [27].

In RA, a case control study reported that individuals with higher DII scores were approximately three times more likely to experience RA [28]. Additionally, a recent cross-sectional study observed that a higher DII was associated with increased RA disease activity [29]. Whilst studies have investigated the association between DII and disease activity and RA risk, limited literature has investigated the effect of improving diet quality on changes in DII in patients with RA and how it impacts nutrient intake patterns and patient reported outcome measures. In previous studies on patients with coronary heart disease (CHD), DII scores significantly improved in patients assigned to a 6-month mediterranean diet compared to those assigned to a low-fat diet [30].

Additionally, improvements in DII scores were significantly associated with reductions in inflammatory markers including interleukin-6 in patients with CHD [31].

Despite the evidence supporting the benefits of improving dietary quality in patients with CHD, research in RA remains limited, revealing a clear gap in current knowledge.

Furthermore, little is known about how changes in DII influence patient outcome measures with one cross-sectional study reporting no significant associations between DI and disease impact or functional impairment [27].

The primary outcomes of the MEDRA randomised controlled trial have been reported elsewhere [23]. That study demonstrated significant improvements in physical function and quality of life following both a Mediterranean Diet and adherence to the Irish Healthy Eating Guidelines, suggesting that enhancing dietary quality benefits adults living with RA [23]. However, the factors underlying these improvements were not explored.

Given the central role of chronic inflammation in RA, and evidence linking the DII with disease risk and severity, the present analysis aimed to evaluate changes in dietary inflammatory potential (DII/e-DII) resulting from both dietary interventions. This analysis also sought to determine whether shifts in DII were associated with nutrient intake patterns and patient-reported outcomes, to provide insights into the anti-inflammatory effects of improved diet quality.

Whilst this present study is based on the findings from the MEDRA study, analyses and results presented herein represent secondary analysis whereby the calculation of DII scores was planned as an exploratory component of the dietary assessment using data collected during the trial, allowing for further examination of the relationship between diet and inflammatory potential beyond the primary clinical outcomes.

## Materials and methods

### Study design and population

This study forms part of a RCT, investigating the effects of a MedDiet and adherence to the Irish national Healthy Eating Guidelines (HEG) in adults with RA – The Mediterranean Diet Intervention in Adults with Rheumatoid Adults (MEDRA) study [23]. This was a 12-week parallel-group, tele-health delivered RCT which analysed two dietary interventions as part of the management of RA [23]. The MEDRA study was designed and conducted in accordance with the Consolidated Standards of Reporting Trials (CONSORT) guidelines [23]. Eligibility criteria for the MEDRA included adults, that were English speaking, with a definite diagnosis of RA according to the 2010 classification criteria by the American College of Rheumatology/European League Against Rheumatism (ACR/EULAR), with access to internet or a mobile phone to receive messages and calls [23, 32]. Individuals were excluded from participation if they were non-English speaking, pregnant, breastfeeding, or had initiated a new dietary program or nutritional supplements within one month prior to the study [23]. Recruitment for the MEDRA study took place between November 2020 and February 2021. Using a two-sample Student’s t-test, with a significance level of 0.05 and 80% power to detect a difference of 0.68 units in the HAQ-DI, the required sample size for the MEDRA study was determined to be 40 participants. This figure was then adjusted to 44 to accommodate a projected 10% dropout rate [25].

A total of 44 participants were recruited, and 40 participants completed the study. The MEDRA study and this extension study were conducted in accordance with the Helsinki Declaration, and ethical approval was granted by the Education and Health Sciences Research Ethics Committee at the University of Limerick and by the Health Service Executive Mid-Western Regional Hospital Research Ethics Committee [23]. Written informed consent was obtained from all participants of the MEDRA study before commencement of the trial [23]. Further details on the MEDRA study have been previously studied [23].

### Data collection

The first teleconsultation was carried out at baseline (0 weeks). Follow-ups were conducted at weeks 3, 6, 9, and 12 via both telephone and video calls. Consultations were carried out by a Registered Dietitian (RD). Demographic data including country of birth, age and sex of participants were collected via open ended questions at baseline teleconsultations. Smoking status, medication and supplement use, dietary food intake and dietary adherence to prescribed diets were also collected and reviewed at each call to monitor changes [23].

### Dietary interventions

The participants of the MEDRA study were randomised into one of two groups, the MedDiet or HEG group [23].

### MedDiet intervention

The MedDiet intervention was derived from the traditional Cretan MedDiet [33]. The dietary guidance within the MedDiet arm recommended a daily consumption of 60-80 ml of extra virgin olive oil, along with at least 2 servings of fruits and 3 servings of vegetables. Participants were advised to consume fish a minimum of 3 times each week and a handful of raw, unsalted nuts on alternate days. The dietary advice was personalised, considering each participant’s food preferences, lifestyle, and routines, with the goal of improving adherence to the principles of the traditional Mediterranean Diet (MedDiet) as reflected in the Mediterranean Diet food pyramid [23]. No specific macronutrient targets were prescribed. Instead, participants were encouraged to achieve recommended servings per day of MedDiet foods emphasising high consumption of vegetables, fruits, wholegrains, legumes, nuts, and olive oil as the principal fat source; moderate intake of fish, poultry, and dairy products; and limited consumption of red and processed meats and foods high in added sugars. Although individualised, the dietary pattern was designed to broadly reflect the typical macronutrient distribution of a traditional Cretan MedDiet approximately 40–45% of total energy from fat (predominantly monounsaturated fatty acids from olive oil), 35–40% from carbohydrates (mainly complex and fibre-rich sources), and 15–20% from protein [33]. This approach supported flexibility and cultural adaptation while maintaining alignment with the core principles of a traditional anti-inflammatory Mediterranean dietary pattern.

### HEG intervention

The HEG intervention was based on the 2016 Irish Healthy Eating Guidelines published by the Department of Health [34]. Participants were advised to consume a daily intake of 5–7 servings of fruits and vegetables, 3 servings of low-fat dairy, 3–5 servings of wholegrain cereals, and 2 servings of lean meat. Advice was also given to participants to reduce their intake of foods high in fat, sugar, and salt to 1 or 2 times per week, along with guidance to consume fats and oils only in small quantities [34]. The aim was to support a balanced dietary pattern consistent with national recommendations, incorporating all five main food groups in appropriate proportions. As with the MedDiet arm, advice was tailored to each participant’s preferences and routines, and no fixed macronutrient targets were prescribed [34].

### Food diaries

Participant dietary intake data including food and beverage intake was collected via 3-day food diaries. Diaries consisted of two weekdays and one weekend day. Diaries were completed at weeks 0, 6, and 12 using the LIBRO mobile application^®^. Nutritional analysis of the diaries was completed by a registered dietitian using Nutritics Software^®^. DII was evaluated at baseline and 12 weeks.

### Dietary adherence

To assess adherence to dietary interventions, data was evaluated at baseline, 6 weeks and 12 weeks using the MEDAS screener and an 11-item healthy eating checklist [23]. The Mediterranean Diet Adherence Score (MEDAS) is a 14-item validated screener designed to assess adherence to a Mediterranean diet with scores ranging from low (between 0 and 5), moderate (scores 6–9) or high (scores 10–14) [23, 35–36]. The HEG group also completed the MEDAS, however an 11-item healthy eating checklist was also developed by researchers to assess compliance with the Irish healthy eating guidelines that participants completed [23].

### Outcome measures

Outcome measures of the MEDRA study were assessed at baseline, week 6 and week 12 [23]. These included changes in physical function and QOL, assessed through two self-administered validated questionnaires – Health Assessment Questionnaire –Disability Index (HAQ-DI) and the Rheumatoid Arthritis Quality of Life (RAQoL) questionnaire [37, 38]. Lower scores were indicative of better physical function and QOL. Secondary outcome measures included physical activity assessed by the validated yale physical activity survey (YPAS), anthropometric measures including weight, and patient perceived pain and general health assessed by the HAQ-DI [37, 39].

### Deriving DII and energy-adjusted DII (e-DII)

DII was calculated based on food diaries collected from MEDRA participants. To calculate the DII of the sample, 39 out of a possible 45 food parameters were included (energy, total fat, polyunsaturated fat, monounsaturated fat, trans fat, saturated fat, protein, carbohydrate, cholesterol, fibre, caffeine, alcohol, iron, magnesium, selenium, thiamine, vitamin A, vitamin C, vitamin D, vitamin E, zinc, vitamin B12, vitamin B6, carotene, folate, niacin, riboflavin, omega-3 fatty acids, omega-6 fatty acids, water, garlic, ginger, onion, saffron, turmeric, green/black tea, pepper, thyme/oregano, and rosemary). Participants beverage intake as reported in the food diaries and participants use of dietary supplements was also factored into the DII and e-DII calculation. To account for the relationships between energy and nutrient intakes, energy-adjusted DII was calculated (38 food parameters employed; energy as the dominator). For certain parameters, such as garlic and onion, it was necessary to estimate the quantities consumed using standard food recipes, as their amount in composite dishes was not clearly described in the food diaries available. The same food recipe was used across each food diary. The calculation of DII and e-DII scores were carried out by Connecting Health Innovations.

### Use of tools to minimise bias

To minimise bias during the group allocation process, participants were randomly allocated to the MD or HEG intervention group with randomisation completed by a statistician who was not involved in the study [25]. To further reduce bias, data was pseudonymised before analysis. All assessments were undertaken by a registered dietitian (RD) and PhD student unblind to patient allocation with a focus on maintaining consistency and implementing standardised protocols to ensure the outcomes of the study were evaluated objectively [23]. This included the use of validated and reliable questionnaires to assess relevant outcome measures, including the Health Assessment Questionnaire, RaQOL, and YPAS, where appropriate. Furthermore, the RD provided clear instructions to MEDRA participants to ensure accurate recording of food and beverage intake and ensure a clear understanding of portion sizes of commonly consumed foods. This approach ensured that the food diaries could be analysed effectively in subsequent evaluations [23].

### Data analysis

Data was analysed using IBM SPSS statistics version 29.0 (IBM Corp.) software for Windows. Normality of variables was assessed using the Shapiro-Wilk test. Data from categorical variables are presented as frequencies and percentages. Continuous variables are presented as mean ± standard deviation. The student’s independent t-test was employed for data comparison of mean scores between two groups for normally distributed continuous variables, with the Mann-Whitney U test used for non-parametric alternatives. The chi-square test was used to assess differences in categorical variables. A paired samples t-test was performed to compare the mean DII and e-DII scores between diet groups pre to post 12-week dietary intervention. Change in DII and e-DII scores between diet groups post-intervention was investigated using an independent t-test. The sample was further categorised into anti-inflammatory and pro-inflammatory groups based on baseline e-DII scores, irrespective of the assigned diet group. The student’s independent t-test was performed to compare mean scores data between the two groups for normally distributed continuous variables. The Mann-Whitney U test was used for non-parametric alternatives, and the chi-square test was employed to compare categorical variables. No multivariable adjustment was performed, however, random assignment to diet groups during the MEDRA study was intended to reduce confounding [25].

Tertiles of change in participant e-DII scores were created. One-way ANOVA was used to assess differences in patient-reported outcome measures (PROMs) across the tertiles for normally distributed data. The Kruskal-Wallis test was used for non-parametric variables. Correlation analysis of 12-week change in e-DII score was performed using Pearson product-moment correlation used for parametric variables and Spearman’s rank order correlation for non-parametric variables. The one-way ANOVA and Kruskal-Wallis test was also used to investigate the change in nutrient intakes across tertiles of post-intervention change in e-DII score. Post hoc tests were employed.

Statistical significance was set at *p* < 0.05, with the exception of when a Bonferroni correction was utilised (*P* < 0.017).

## Results

### Baseline characteristics and dietary intake

Baseline characteristics of participants are presented in the MEDRA study [25]. The majority of study participants were female subjects (87.5%), born in Ireland (92.5%) and the mean ± SD age was 47.5 ± 10.9 years old. Most participants (97.5%) were using disease-modifying anti-rheumatic drugs (DMARDS) to help with the management of their RA. The average general health score for total participants was 47.9 ± 27.7. The average pain score was 42.6 ± 25.7. The average disease duration was 9.5 ± 9.6 years.

There was no significant difference in baseline characteristics between diet intervention groups, with the exception of the HAQ-DI score (*p* = 0.006), which was significantly lower in the MedDiet group (0.9 ± 0.5) compared to the HEG group (1.4 ± 0.7) [25].

The majority of participants (*n* = 28) had a pro-inflammatory e-DII score at baseline as determined by DII and eDII scores. As was previously reported in the MEDRA RCT [23], at baseline, the MedDiet group had a low adherence to the MedDiet intervention (Mean MEDAS score 5.9 ± 2.5 out of 14), whilst the HEG group had an average adherence score of 6.3 ± 1.7 out of 11 to the Irish HEG. The total cohort was pooled to assess the total adherence to the MedDiet with an average MEDAS score of 5.1 ± 2.3 out of 14 [23]. Furthermore, at baseline, energy intake did not significantly differ between groups (MedDiet 6725.84 ± 1052.89 KJ) (HEG 6200.66 ± 2124.85 kJ). There were no significant differences in micro- or macronutrient intake at baseline. On average, protein, carbohydrates, and fat contributed 19.5 ± 4.9%, 42.6 ± 8.4%, and 38.9 ± 7.2% of total energy intake for the total cohort, respectively. The mean alcohol and water consumption for the total cohort was 2.7 ± 6.2 g and 1498.1 ± 870.3 g, respectively. Average caffeine intake was 89.9 ± 87.8 mg across the cohort, with the HEG group reporting a significantly higher intake compared to the MedDiet group at baseline (118.8 ± 86.7 mg vs. 61.2 ± 81.1 mg, *p* < 0.05) [23].

Additionally, in the analysis of the consumption of food groups participants in the MedDiet group reported a significantly higher intake of fish compared to the HEG group (1.1 ± 0.8 vs. 0.1 ± 0.2 servings, *p* = 0.00). In the analysis of the pooled cohort (*n* = 40), the mean daily intake was 6.8 ± 1.5 servings from the grains group, 1.1 ± 0.8 servings of fruit, 2.0 ± 1.6 servings of vegetables, 0.8 ± 0.9 servings of extra virgin olive oil, 0.3 ± 0.4 servings of fish, and 1.0 ± 0.7 servings of red and processed meats [23].

### Changes in outcome measures

Detailed information regarding significant changes in outcome measures have been published elsewhere [23]. In summary, both MedDiet and HEG groups reported significant improvements in physical function (*p* < 0.001) and quality of life (*p* < 0.001) [23]. Participants in the MedDiet group also improved physical activity levels (*p* = 0.01). Both groups reported significant reductions in weight (MedDiet − 0.6 kg, *p* < 0.001) (HEG − 0.9 kg, *p* = 0.004) and BMI (MedDiet: 26.1 ± 5.5 to 25.9 ± 5.5 Kg/m^2^, *p* = 0.001) (HEG: 27.2 ± 4.9 to 26.9 ± 4.7 Kg/m^2^, *p* = 0.002) [23].

### DII and e-DII scores following dietary intervention

The mean baseline DII and e-DII for the cohort were 2.23 ± 2.19 and 0.99 ± 2.37, respectively. There was no significant difference between mean DII and e-DII scores of the two diet groups at baseline (DII MedDiet: 1.79 ± 2.26 vs. HEG: 2.67 ± 2.08, *P* = 0.207; e-DII, MedDiet: 0.79 ± 2.60 vs. HEG: 1.20 ± 2.16, *P* = 0.588).

Table 1 demonstrates that there were significant improvements in e-DII for both diet groups (*p* < 0.001) following the 12-week intervention. In the assessment of DII, there was a significant improvement in the MedDiet group (*p* = 0.022), but not for the HEG groups (*p* = 0.061). Table 2 presents the degree of change in DII and e-DII scores post dietary intervention.

**Table 1:** DII and e-DII post 12-week dietary intervention for the MEDRA participants

		All		MedDiet *n* = 20		HEG *n* = 20	
		DII	e-DII	DII	e-DII	DII	e-DII
*Pre-intervention*							
Mean		2.23	0.99	1.79	0.79	2.68	1.2
SD		2.19	2.37	2.26	2.6	2.08	2.16
*Post-intervention*							
Mean		0.77	− 1.3	0.03	− 1.84	1.5	− 0.76
SD		2.3	1.8	1.77	1.54	2.56	1.91
*p*-value		0.003	< 0.001	0.022	0.002	0.061	0.004

MedDiet, Mediterranean Diet; HEG, Healthy Eating Guidelines; DII, Dietary Inflammatory Index; e−DII, energy−adjusted Dietary Inflammatory Index; SD, Standard Deviation

All data are presented as mean ± SD. P−values were derived by a paired t−test and display significant and non−significant differences between pre and post mean values

**Table 2:** Degree of change in DII and e-DII scores post dietary intervention

	MedDiet *n* = 20	HEG *n* = 20			
	Mean	SD	Mean	SD	*p*-value
Change in DII	− 1.76	3.14	− 1.18	2.64	0.527
Change in e-DII	− 2.63	3.22	− 1.96	2.69	0.483

MedDiet, Mediterranean Diet; HEG, Healthy Eating Guidelines; DII, Dietary Inflammatory Index; e−DII, energy−adjusted Dietary Inflammatory Index; SD, Standard Deviation. All data are presented as mean ± SD. Negative values reflect anti−inflammatory scores, while positive values reflect pro−inflammatory scores. P−values were derived by an independent t−test

The observed change in DII and e-DII was greater in the MedDiet group (− 1.76 ± 3.14, − 2.63 ± 3.22) compared to the HEG group (− 1.18 ± 2.64, − 1.96 ± 2.69), but this greater change observed was not statistically significant (p = > 0.05).

### Anti-inflammatory versus pro-inflammatory e-DII

Baseline characteristics of the participants according to their pro and anti-inflammatory e-DII scores at baseline is outlined in Table 3. Participants with a pro-inflammatory e-DII score at baseline (*n* = 28) had a greater mean weight and BMI, increased patient-reported pain scores matched with a lower overall health status score, compared to participants with an anti-inflammatory e-DII score. However, these differences were not statistically significant between the diet groups.

**Table 3:** Characteristics of participants according to baseline anti (−) or pro (+) inflammatory e-DII scores

Variable	e-DII score (-) anti-inflammatory (*n* = 12)	e-DII score (+) pro- inflammatory (*n* = 28)	*p*-value
Age (years)	47.7 ± 11.17	47.5 ± 11.0	0.570
Females, n (%)	11 (91.7)	24 (85.7)	0.602
*Anthropometry*			
Height (m)	1.7 ± 0.16	1.6 ± 0.1	0.694
Weight (kg)	69.8 ± 16.1	73.0 ± 14.3	0.538
BMI (kg/m^2^)	25.6 ± 5.8	27.1 ± 4.8	0.375
Country of birth (Ireland), n (%)	11 (91.7)	26 (92.9)	0.363
Marital status, n (%)			0.143
Single	1 (8.3)	5 (17.9)	
Married	7 (58.3)	16 (57.1)	
Divorced	2 (16.7)	0 (0)	
Living with a partner	2 (16.7)	7 (25)	
Education, n (%)			0.850
Secondary school	1 (8.3)	2 (7.1)	
Apprentice/trade	0 (0)	3 (10.7)	
Certificate/diploma	3 (25)	7 (25)	
Bachelor’s degree	5 (41.7)	9 (32.1)	
Master’s degree	2 (16.7)	6 (21.4)	
Doctoral	1 (8.3)	1 (3.6)	
Employment, n (%)			0.670
Unemployed	1 (8.3)	5 (17.9)	
Full-time	8 (66.7)	17 (60.7)	
Part-time	1 (8.3)	4 (14.3)	
Retired	2 (16.7)	2 (7.1)	
Smoking status, n (%)			0.274
Non-smoker	11 (91.7)	19 (67.9)	
Former smoker	1 (8.3)	8 (28.6)	
Current smoker	0 (0)	1 (3.6)	
Disease duration (years)	10.2 ± 10.4	9.1 ± 9.5	0.763
DMARD use, n (%)	12 (100)	27 (96.4)	
Patient-reported pain levels [0-100]	35.0 ± 18.7	45.9 ± 27.9	0.247
Patient-reported overall health status score [0-100]	49.2 ± 26.7	47.4 ± 28.5	0.873
RAQoL score	10.7 ± 9.5	10.7 ± 6.3	0.673
*YPAS- summary index score*			
Sitting baseline	2.5 ± 0.5	2.5 ± 1.1	0.550
Standing baseline	7.8 ± 3.7	8.6 ± 4.3	0.610
Moving baseline	9.0 ± 3.4	8.9 ± 3.8	0.896
Leisurely walking baseline	20.0 ± 12.7	22.3 ± 16.1	0.694
Vigorous activity baseline	16.3 ± 16.4	15.7 ± 17.1	0.942
Summary index baseline	55.5 ± 28.2	57.6 ± 31.2	0.805
HAQ-DI score	1.0 ± 0.7	1.2 ± 0.6	0.389

e−DII, energy−adjusted DII; BMI, Body Mass Index; DMARD, Disease modifying antirheumatic drugs; RAQoL: Rheumatoid Arthritis Quality of Life; YPAS, Yale Physical Activity Survey

All data are presented as mean ± SD and n (%). p−values were derived from the Student’s t−test for normally distributed continuous variables, the Mann−U Whitney was used for non−parametric alternatives, and the chi−square test for categorical variables

### Tertiles of change in e-DII score

Participants, irrespective of their diet group, were classified into tertiles of 12-week change in e-DII score (Table 4); tertile 1: ≤-3.23 (T1, *n* = 14), tertile 2: -3.23- -0.69 (T2, *n* = 12) and tertile 3: -0.68+(T3, *n* = 14). The most anti-inflammatory e-DII scores are represented in tertile 1, with the most pro-inflammatory e-DII scores represented in tertile 3. PROMs were assessed, with no significant differences observed for variables across the three tertiles. However, participants categorised into tertile 1 had a lower HAQ-DI and patient-reported overall health score, indicating better physical function and better health status. These scores increased across the tertiles, demonstrating a poorer profile of patient outcome measures. Similar trends were observed for YPAS summary index scores for sitting and leisurely walking. Partial correlation coefficients between change in e-DII tertiles are included in Table 5. Correlation coefficients were generally low, with no statistically significant correlations observed. A weak positive correlation was observed for HAQ-DI with 12-week change in e-DII score but was not significant (*p* = 0.075).

**Table 4:** Patient reported outcome measures at 12 weeks, post-dietary intervention, across tertiles of change in e-DII score, with upper and lower limits of each variable measure

Variable measures	T1(≤ − 3.23) *n*=14	UV, LV	T2(− 3.23–0.69) *n*=12	UV, LV	T3(− 0.68+) *n*=14	UV, LV	*p*-value
*Anthropometry*							
Weight (kg)	71.8 ± 14.2	49.0, 94.5	69.4±15.2	48,102	72.5±15.2	50.0,96.0	0.87
BMI (kg/m^2^)	26.7 ± 3.9	21.2,34.8	24.7±4.4	19.3,32.0	27.5±6.5	16.9, 37.7	0.4
Patient-reported overall health status score [0-100]	21.4 ± 26.4	0,90	28.3±28.1	0,75	36.6±33.7	0,95	0.5
RAQoL YPAS-summary index score	4.6 ± 6.0	0,20	6.1±6.0	0,20	7.1±5.9	1,16	0.33
Sitting	3.2 ± 2.2	1,8	3.0±1.4	1,6	2.4±1.8	1,8	0.19
Standing	9.4 ± 6.2	0,24	7.2±5.0	0,15	8.4±3.2	3,15	0.58
Moving	9.4 ± 3.7	3,15	9.0±3.8	3,15	9.0±3.3	3,15	0.93
Leisurely walking	27.7 ± 15.9	0,48	24.3±15.1	0,48	20.6±14.3	0,48	0.54
Vigorous activity	20.7 ± 19.9	0,60	21.1±19.5	0,48	12.1±14.9	0,40	0.34
Summary index	70.5 ± 30.5	15, 129	64.6±31.3	30,124	52.5±27.1	8,97	0.37
HAQ-DI	0.7 ± 0.7	0,2.5	0.8±0.5	0,1.5	0.9±0.5	0.3, 2.0	0.2

Participants were distributed into tertiles based on their 12-week change in e-DII score

BMI, Body Mass Index; RAQoL: Rheumatoid Arthritis Quality of Life; YPAS, Yale Physical Activity Survey; HAQ-DI, Health Assessment Questionnaire –Disability Index; LV, Lower Value; UV, Upper Value

All data are presented as mean ± SD. p- values were derived from the Kruskal Wallis test/one way ANOVA. R-values are presented as Spearman/Pearson (r)coefficients

**Table 5:** Partial correlation coefficients between change in e-DII tertiles

Variable	Correlation with 12 weeks change in e-DII score (*r*-value)	Significance of correlation (*p*-value)
*Anthropometry*		
Weight (kg)	0.053	0.744
BMI (kg/m2)	0.133	0.413
Patient-reported pain levels [0-100]	0.019	0.907
Patient reported overall health status score [0-100]	0.107	0.51
RAQoL	0.227	0.158
*YPAS-Summary Index Score*		
Sitting	-0.176	0.277
Standing	-0.068	0.678
Moving	-0.107	0.513
Leisurely walking	-0.239	0.138
Vigorous activity	-0.17	0.294
Summary index	-0.265	0.098
HAQ DI Score	0.285	0.075

The differences across the tertiles of change in e-DII scores and changes in nutrient and food group intake are presented in Table 6. Statistically significant differences were observed between the tertiles for intakes of omega-3 (p=0.002), dietary fibre (p=0.008), vitamin A (p=0.003), vitamin E (p=0.001), folic acid (p=0.006), and beta-carotene (p=0.004). The significant differences lay most frequently between tertile 1 and 3. Participants in T1 had the greatest increase in their intake of dietary fibre, vitamin A, vitamin E and beta carotene. T2 had the greatest increase in omega-3 and folic acid intake. Intake of all these nutrients decreased in T3.

**Table 6:** Change in nutrient intakes across tertiles of post-intervention change in e-DII score

Intake change variable	T1 (≤-3.23) *n* = 14	T2 (-3.23- -0.69) *n* = 12	T3 (-0.68+) *n* = 14	*p*-value
*Macronutrients*				
Energy (kcal)	-249.1 ± 623.4	-187.8 ± 437.5	-72.4 ± 383.1	0.636
Carbohydrate (g)	-25.3 ± 47.3	0.5 ± 48.4	-16.7 ± 48.5	0.393
Protein (g)	-2.1 ± 44.3	-1.3 ± 22.5	-1.8 ± 21.8	0.774
Fat (g)	-5.0 ± 36.1	− 17.0 ± 21.2	1.7 ± 24.0	0.249
Trans Fat (g)	− 0.2 ± 0.3	− 0.4 ± 0.5	− 0.1 ± 0.3	0.187
Cholesterol (mg)	− 117.5 ± 163.6	− 71.6 ± 107.8	− 36.2 ± 138.5	0.346
Saturated Fats (g)	− 6.1 ± 7.3	− 9.4 ± 5.7	− 2.8 ± 6.6	0.054
MUFA (g)	5.8 ± 21.6	2.0 ± 11.3	2.3 ± 12.1	0.928
PUFA (g)	4.3 ± 6.9	1.1 ± 5.8	− 0.4 ± 4.7	0.193
Omega− 3(g)	1.4 ± 1.3	1.9 ± 2.2^¥^	− 0.3 ± 0.9^‡^	0.002*
Omega− 6(g)	0.6 ± 3.9	− 2.7 ± 6.3	0.1 ± 3.9	0.191
Dietary Fibre (g)	7.7 ± 6.6	2.2 ± 7.9	− 1.1 ± 6.5^‡^	0.008*
Caffeine (g)	− 0.04 ± 0.1	− 0.1 ± 0.1	− 0.02 ± 0.1	0.539
Alcohol (g)	− 0.7 ± 5.5	2.0 ± 9.1	2.3 ± 9.0	0.454
*Micronutrients*				
Iron (mg)	1.6 ± 6.0	2.9 ± 6.8	− 0.5 ± 5.0	0.354
Vitamin A (µg)	1276.4 ± 1510.7	− 349.7 ± 792.4^†^	− 138.8 ± 776.6^‡^	0.003*
Vitamin E (mg)	5.4 ± 7.1	1.4 ± 5.9	− 1.3 ± 2.8^‡^	0.001*
Vitamin C (mg)	36.9 ± 94.2	85.3 ± 92.1	6.1 ± 92.0	0.226
Vitamin D (µg)	2.0 ± 4.3	3.5 ± 4.6	− 0.6 ± 3.1	0.063
Zinc (mg)	− 1.0 ± 9.8	− 2.0 ± 3.6	− 0.5 ± 2.1	0.144
Vitamin B12 (µg)	− 0.2 ± 4.4	0.9 ± 3.0	− 0.4 ± 1.3	0.329
Vitamin B6 (mg)	0.24 ± 1.1	0.6 ± 0.6	− 0.2 ± 0.7	0.077
Folic Acid (µg)	84.6 ± 134.0	87.4 ± 66.2^¥^	− 675.8 ± 2365.6	0.006*
Beta Carotene (µg)	7140.6 ± 7565.4	− 1288.9 ± 4666.0^†^	− 1292.6 ± 4686.3^‡^	0.004*
Niacin (mg)	5.4 ± 21.2	1.1 ± 17.8	− 4.4 ± 10.4	0.244
Riboflavin (mg)	0.2 ± 0.6	1.1 ± 3.8	− 0.3 ± 0.5	0.050
Thiamine (mg)	0.1 ± 0.7	0.3 ± 0.7	− 0.2 ± 1.4	0.645
Selenium (µg)	5.4 ± 37.7	16.5 ± 21.0	− 2.0 ± 20.1	0.251
Magnesium (mg)	78.0 ± 144.3	7.1 ± 104.0	− 17.1 ± 87.8	0.258
Garlic (g)	1.3 ± 5.9	− 2.2 ± 8.1	2.5 ± 4.6	0.307
Ginger (g)	0.5 ± 2.0	0.0 ± 0.0	0.7 ± 6.0	0.826
Turmeric (mg)	0.9 ± 2.5	0.6 ± 1.5	− 75.0 ± 280.6	0.372
Black/Green Tea (g)	1.0 ± 4.0	0.7 ± 3.7	− 3.4 ± 8.3	0.314
Pepper (g)	0.1 ± 0.3	− 0.0 ± 0.2	− 0.1 ± 0.3	0.741
Thyme/Oregano(mg)	7.2 ± 26.7	− 0.1 ± 0.1	− 128.3 ± 481.1	0.232
Rosemary (mg)	0 ± 0.1	0.0 ± 0.2	0.0 ± 0.0	1.0
*Food group (serves/d)*				
Vegetables	1.9 ± 1.0	2.3 ± 1.4	1.6 ± 1.5	0.471
Fruit	1.0 ± 0.8	0.6 ± 0.6	1.1 ± 0.9	0.181
Grains	− 0.3 ± 1.6	1.6 ± 2.3	0.7 ± 1.9	0.066
Legumes	0.9 ± 0.5	0.6 ± 0.3	0.7 ± 1.2	0.118
Dairy	0.6 ± 0.6	0.6 ± 0.5	0.8 ± 0.6	0.587
Poultry	0.4 ± 0.7	0.0 ± 0.7	0.3 ± 0.9	0.649
Red/Processed Meats	− 0.6 ± 0.8	− 0.5 ± 0.8	− 1.0 ± 0.7	0.249
Fish	0.4 ± 0.4	0.5 ± 0.5	0.5 ± 0.5	0.897
Nuts, Seeds	0.3 ± 0.4	0.4 ± 0.4	0.2 ± 0.2	0.230
EVOO (Tbsp./ day)	1.3 ± 1.8	1.6 ± 1.7	1.0 ± 1.6	0.716

PUFA, Poly−unsaturated fatty acids; MUFA, Mono−unsaturated fatty acids; EVOO, Extra virgin olive oil; Tbsp., Tablespoon

All data are presented as mean ± SD. p−values were derived from the Kruskal Wallis test/One way ANOVA

*Significant difference lies between these two groups at *p* <0.05

†Significant difference between tertile 1 and 2, P <. 0.017 (Bonferroni correction)

‡ Significant difference between tertile 1 and 3, *P* < 0.017 (Bonferroni correction)

¥ Significant difference between tertile 2 and 3, *P* < 0.017 (Bonferroni correction)

## Discussion

The present study aimed to investigate the effects of two dietary interventions (MedDiet and HEG) on changes in dietary inflammatory potential (DII/e− DII) and to explore the relationship between change in DII and e− DII with nutrient intake patterns and patient−reported outcomes.

Several notable findings have emerged. Firstly, the majority of participants with RA who took part in this study had a pro− inflammatory DII score at baseline. Post− intervention, participants significantly improved e− DII scores by adhering to a MedDiet or the Irish HEG. Significant differences were observed across the tertiles of change in e− DII scores with change in nutrient and food group intake highlighting that participants with the lowest e− DII scores (anti− inflammatory) had significantly increased intakes of dietary fibre, vitamin A, vitamin E, and beta carotene after improving diet quality following a 12− week dietary intervention.

Results from this study demonstrate that pre− intervention, both dietary groups were consuming a relatively pro− inflammatory dietary pattern. Previous studies have reported similar pro− inflammatory dietary patterns in people with RA including a study observing the dietary patterns of participants that took part in the National Health and Nutrition Examination Survey (NHANES) [40]. In this study, participants with RA had a mean DII score of 3.26 ± 1.76, reflective of being a pro− inflammatory dietary pattern [40].

There is a large body of literature investigating dietary patterns and the risk of RA [41, 42]. Previous studies have reported that the consumption of a pro− inflammatory diet, namely a diet similar to that of the Western diet is thought to increase RA risk by increasing inflammation and increasing the risk of obesity and insulin resistance, which are risk factors for RA development [43]. This has been observed in a recent case control study [44] whereby higher adherence to a Western dietary pattern (compared to lower adherence) was positively associated with RA risk (multivariable-adjusted OR tertile 3 vs. 1:1.95; 95% CI: 1.09–3.92; *p*‐trend: 0.046) [44]. Additionally, certain elements of the Western Diet including sugar sweetened beverages and red and processed meats have been extensively studied because of their role in increasing the risk of RA, with results from the Nurses Health prospective study highlighting that women who consumed greater than 1 serving of a sugar sweetened beverage in a day had a 63% increased risk of RA development compared to women who consumed less than one sugar sweetened beverage (HR: 1.63; 95% CI: 1.15, 2.30; *P−* trend = 0.004) [45].

For individuals living with RA, adhering to a Western dietary pattern can contribute to the inflammatory response and worsen RA associated symptoms [46–47]. Red meat is a significant source of arachidonic acid (AA), contributing to inflammation, joint damage and pain [46]. Additionally, meat and full− fat dairy products are rich in saturated fats, which may contribute to inflammation induced by adipose tissue [47].

Results from the recently published Irish National Adult Nutrition Survey (NANS) show that the general Irish population follow a typically Western diet [48]. In particular, the NANS reported that ‘top shelf foods’ (foods high in trans and saturated fat, sugar and sodium) constituted one fifth of total calories consumed [48]. Additionally, overall sodium, sugar and saturated fat intake were higher than recommendations for the Irish adult population with reported low intakes of fibre, fruit and vegetables [48]. This pro− inflammatory dietary pattern, similar to what has been observed by participants in this study, residing in Ireland, may pose potential health risks to the Irish adult population and contribute further to inflammatory type phenotypes [44].

Despite this pro− inflammatory state at baseline, adhering to a high− quality diet, such as the MedDiet and nationally promoted HEG, improved the dietary inflammatory profiles across the 12− week intervention in participants with RA in this study. However, improvement in DII and e− DII were significantly greater in the MedDiet group compared to the HEG group. This finding is clinically relevant, as it demonstrates that participants improved their DII and e− DII scores by improving their overall diet quality. Similar to this present study, in patients with coronary heart disease, studies have reported the effects of a MedDiet and low− fat dietary intervention on improving DII and e− DII scores, whereby participants adhering to a MedDiet had significantly reduced DII scores, while no change was observed in the low− fat diet group [49]. While these findings were not RA− specific, they provide comparisons in a population group with a disease with a similar underlying inflammatory pathogenesis.

Several studies have demonstrated the benefits of the mediterranean diet in improving RA related outcome measures including the MEDRA study and a recently conducted study reporting that a greater adherence to the MedDiet intervention was associated with significant improvements in disease activity scores (DAS− 28) (*p* < 0.001), BMI (*p* < 0.001) and inflammatory markers including CRP (*p* = 0.030) and ESR (*p* < 0.001) [23, 50]. Research has indicated that the beneficial effects of this dietary pattern may be attributed to several key components of this dietary pattern including oily fish, whole grains, fruits, vegetables, and olive oil [13].

Certain whole grains, along with walnuts, flaxseeds and oily fish, are excellent sources of omega− 3 polyunsaturated fatty acids, including eicosapentaenoic acid (EPA) and docosahexaenoic acid (DHA). These fatty acids can lead to a reduction in arachidonic acid (AA) content in cell membranes, subsequently decreasing the production of pro− inflammatory cytokines derived from AA and helping to mitigate tissue damage and inflammatory− related pain [13, 18, 21]. Additionally, the moderate consumption of olive oil has been associated with health benefits for patients with rheumatoid arthritis (RA), largely attributable to its content of the monounsaturated fatty acid (MUFA) oleic acid [18, 51]. Recent studies, including the TOMORROW study highlighted that MUFA intake was significantly lower in the RA group (*n* = 208) versus the control group (individuals that did not have RA, *n* = 205) [51]. Increased MUFA intake was also significantly associated with improved RA outcome measures as indicated by lower DAS− 28 scores (*R* = 0.180, *P* = 0.01) [51].

Another key component of the Mediterranean diet includes fruits and vegetables which are rich in antioxidants, fibre and essential minerals, which may help counteract the effects of free radicals and pro− inflammatory cytokines that contribute to tissue damage in RA [52]. This has been observed in previous research whereby a greater intake of fruit and vegetables was associated with lower disease activity in patients with RA (*n* = 441) (ρ = −0.11, *p* < 0.01) [52].

The Irish HEG has many similarities to the advised MedDiet including the promotion of a large consumption of fruit and vegetables, fibre rich foods and limiting the consumption of red meat and processed foods [34]. In this present study, the HEG intervention was also effective in reducing e− DII scores. The similarities between MedDiet and HEG may account for the beneficial effects observed in this group, compared to the non− significant findings observed with a diet purely focused on low− fat dietary strategies. Whilst the mediterranean diet has proven benefits in RA populations, a qualitative analysis of the MEDRA study found that some individuals assigned to the MedDiet group found accessing suitable foods/meals consistent with the principles of the MedDiet challenging [53]. Additionally, participants also commented that the MedDiet appeared to be more expensive than their normal dietary pattern [53]. These barriers may make it difficult for individuals with RA to adhere to a Mediterranean diet for a long period of time. Based on the positive results observed by participants assigned to the HEG group, adhering to nationally promoted guidelines may be a more feasible and acceptable dietary approach for individuals to follow, whilst achieving the same positive results.

Another important finding of the current study is that the intake of various nutrients improved e− DII scores across the tertiles. Participants with greater anti− inflammatory e− DII scores at post intervention, irrespective of diet group, had significantly greater intakes of dietary fibre, vitamin A, vitamin E, and beta− carotene. The link between improved DII score and increased fibre intake in RA patients has been demonstrated in various studies [27, 54]. Previous studies have observed that participants had statistically lower fibre intakes in the fourth quartile compared to the first quartile of DII scores [54]. Similar to this study, intakes of nutrients such as energy, protein, and carbohydrates were not statistically different across the quartiles [54]. A previous cross− sectional study observed that the fibre intake was significantly greater in participants with the highest adherence to the MedDiet, and was the single nutrient found to be different between MedDiet adherence groups [27]. Additionally, in a previous study based on participants with coronary heart disease, participants who had experienced a previous cardiac event and had the largest decline in DII scores had increased intakes of omega− 3, fibre, and vitamin E, and folate [49]. In a prospective study examining MedDiet, DII, and lung cancer incidence, anti− inflammatory DII scores were associated with a higher intake of fruits, vegetables, fish, wholemeal bread, and legumes [55]. These foods are associated with the nutrients that were statistically significant across e− DII tertiles of nutrient intake change in the present study. Fibre, omega 3, vitamin E and vitamin A are all key components of a high− quality diet, particularly of the MedDiet and may work in synergy given that they have been extensively researched for their anti− inflammatory properties [56–57].

Although the MEDRA study was not specifically designed as a weight loss intervention, both dietary groups reported statistically significant weight reductions following the intervention [23]. Ongoing research is exploring the factors that influence energy metabolism in individuals with RA and the most effective ways to support those experiencing inflammation and pain [58]. The inflammatory processes associated with RA cause certain immune cells, such as macrophages and neutrophils, to modify their energy metabolism, resulting in an elevated production of reactive oxygen species that can lead to tissue damage and pain [59]. Being overweight has been linked to increased levels of inflammatory markers, which can further disrupt energy metabolism and exacerbate RA symptoms, including pain [60, 61].

Research indicates that weight loss and effective management of energy intake can contribute to reduced inflammation and pain [60, 61]. This finding suggests that not only did participants in the MEDRA study reduce the inflammatory potential of their diets, but consuming a healthy balanced diet also resulted in significant weight loss over a 12− week period. This weight reduction may positively impact energy metabolism and further alleviate pain and inflammation. Future research may investigate how calorie management can enhance the regulation of inflammatory responses and improve RA symptoms.

It is important to acknowledge the limitations of the current study. The assessment of inflammatory markers such as CRP and ESR would provide an objective measure of inflammation and RA activity. Additionally, the sample size of the cohort (*n* = 40) must be considered. Although not statistically significant, participants with greater anti− inflammatory e− DII observed improvements in patient reported outcome measures (PROMS) at the end of the 12− week intervention. It would be valuable to observe if these results would be significant in a larger diverse population group similar to previous studies with larger powered population sizes ranging from 120 to 184 participants [27, 29, 57]. Additionally, the use of objective measures to assess RA outcome measures such as biochemical data may strengthen the significance of results.

Despite these limitations, the study has several strengths. This study utilised 39 out of 45 parameters for DII calculation, compared to other studies which employed 26–34 parameters [27, 50, 54]. Another strength is the RCT design of the MEDRA study [23]. Furthermore, the study employed the use of robust parameters to assess data collected on PROMs. The HAQ− DI is a validated, gold− standard assessment tool, while the RAQoL questionnaire to assess QOL is also validated [37−39]. The use of a 3− day food diary can also be listed as a strength, due to its validation by a registered dietitian, who also provided dietary counselling throughout the intervention. This method of dietary assessment is more robust for DII calculation compared to food frequency questionnaires, which are a common dietary assessment method for DII calculation [57].

## Conclusion

This study demonstrates that adhering to a high− quality diet, such as the MedDiet or the Irish HEG, can improve e− DII scores in RA patients. However, this finding is not associated with a significant change in patient reported outcome measures including physical function and quality of life. Participants in the most anti− inflammatory e−.

DII tertile had greater intakes of omega− 3, dietary fibre, vitamin A, vitamin E, folic acid, and beta− carotene across the intervention period. Larger powered studies with objective measures are needed to further investigate the effects of an anti− inflammatory diet on physical function and QOL in adults with RA.

## Abbreviations

BMI: Body mass index
RAQoL: Rheumatoid arthritis quality of life
YPAS: Yale physical activity survey
HAQ-DI: Health assessment questionnaire-disability index
LV: Lower value
UV: Upper value
